# Supplementary material for: High mortality among kidney transplant recipients diagnosed with coronavirus disease 2019: Results from the Brazilian multicenter cohort study
Source: PLoS One. 2021 Jul 28;16(7):e0254822. doi: 10.1371/journal.pone.0254822 (PMC8318290; doi:10.1371/journal.pone.0254822)
Supplement: S1 Table — Footnote: In all cases where informed consent was obtained, it was written. In some specific cases, the informed consent was waived by the local ethics committees. Legend: IRB, Institutional Review Board; PI, principal investigator. (DOCX) [file pone.0254822.s001.docx]

S1 Table. Institutional Review Boards (IRB)

| **SITE / IRB** | **PI** | **Approval Number** |
| --- | --- | --- |
| INVITARE PESQUISA CLINICA AUDITORIA E CONSULTORIA LTDA | José Osmar Medina Pestana | 3.963.792 |
| IRMANDADE DA SANTA CASA DE MISERICORDIA DE PORTO ALEGRE - ISCMPA | Valter Duro Garcia | 4.076.184 |
| HOSPITAL GERAL DE FORTALEZA | Tainá Veras De Sandes Freitas | 4.079.952 |
| HOSPITAL UNIVERSITÁRIO WALTER CANTÍDIO DA UNIVERSIDADE FEDERAL DO CEARÁ / HUWC - UFC | Claudia Oliveira | 4.281.932 |
| FACULDADE DE MEDICINA DE SÃO JOSE DO RIO PRETO- FAMERP - SP | Mario Abbud Filho | 4.062.159 |
| SOCIEDADE BENEF ISRAELITABRAS HOSPITAL ALBERT EINSTEIN | Lúcio Roberto Requião Moura | 4.229.281 |
| HOSPITAL UNIVERSITÁRIO DA UNIVERSIDADE FEDERAL DO MARANHÃO / HU | Teresa Cristina Alves Ferreira | 4.357.647 |
| HOSPITAL DE CLÍNICAS DE PORTO ALEGRE DA UNIVERSIDADE FEDERAL DO RIO GRANDE DO SUL | Roberto Ceratti Manfro | 4.081.653 |
| HOSPITAL UNIVERSITÁRIO ONOFRE LOPES DA UNIVERSIDADE FEDERAL DO RIO GRANDE DO NORTE - HUOL/UFRN | Kellen Micheline Alves Henrique Costa | 4.074.811 |
| UNIVERSIDADE REGIONAL DE BLUMENAU- FURB | Denise Rodrigues Simão | 4.416.524 |
| UNICAMP - CAMPUS CAMPINAS | Marilda Mazzali | 4.184.616 |
| SANTA CASA DE MISERICÓRDIA DE JUIZ DE FORA/MG | Gustavo Fernandes Ferreira | 4.118.980 |
| UNESP -FACULDADE DE MEDICINA DE BOTUCATU | Luis Gustavo Modelli De Andrade | 4.342.044 |
| INSTITUTO HOSPITAL DE BASE DO DISTRITO FEDERAL -IHBDF | Viviane Brandao Bandeira De Mello Santana | 4.154.446 |
| HOSPITAL MUNICIPAL SÃO JOSÉ/ HMSJ JOINVILLE/ SC | Luciane Mônica Deboni | 4.073.338 |
| HOSPITAL DAS CLÍNICAS DA FACULDADE DE MEDICINA DE RIBEIRÃO PRETO DA USP | Elen Almeida Romão | 4.323.540 |
| CASA DE SAÚDE SANTA MARCELINA | Juliana Aparecida Zanocco | 4.478.600 |
| UNB - FACULDADE DE MEDICINA DA UNIVERSIDADE DE BRASÍLIA | Gustavo Guilherme Queiroz Arimatea | 4.112.218 |
| UNIRIO - HOSPITAL UNIVERSITÁRIO GAFFREE E GUINLE / HUGG- UNIRIO | Deise Rosa De Boni Monteiro De Carvalho | 4.431.718 |
| PONTIFÍCIA UNIVERSIDADE CATÓLICA DO PARANÁ - PUC/ PR | Alexandre Tortoza Bignelli | 4.407.658 |
| HOSPITAL BENEFICÊNCIA PORTUGUESA DE SÃO PAULO | Irene De Lourdes Noronha | 4.194.650 |
| UERJ - HOSPITAL UNIVERSITÁRIO PEDRO ERNESTO/ UNIVERSIDADE DO ESTADO DO RIO DE JANEIRO | Suzimar Da Silveira Rioja | 4.299.892 |
| HOSPITAL FELÍCIO ROCHO/MG | Rafael Lage Madeira | 4.079.485 |
| HOSPITAL MÁRCIO CUNHA - FUNDAÇÃO SÃO FRANCISCO XAVIER | Carlos Alberto Chalabi Calazans | 4.305.309 |
| SANTA CADA DE MISERICORDIA DE SÁO PAULO | Patricia Malafronte | 4.463.388 |
| UFPE - HOSPITAL DAS CLÍNICAS DA UNIVERSIDADE FEDERAL DE PERNAMBUCO - HC/UFPE | Filipe Carrilho De Aguiar | 4.229.065 |
| HOSPITAL PARANAENSE DE OTORRINOLARINGOLOGIA - IPO | Fabiana Loss De Carvalho Contieri | 4.380.026 |
| UFJF - HOSPITAL UNIVERSITÁRIO DA UNIVERSIDADE FEDERAL DE JUIZ DE FORA - MG | Hélady Sanders Pinheiro | 4.156.755 |
| UNIVERSIDADE DO VALE DO ITAJAÍ-UNIVALI / SANTA CATARINA | Andre Barreto Pereira | 4.467.401 |
| HOSPITAL ALEMÃO OSWALDO CRUZ - SP | David José De Barros Machado | 4.238.473 |
| FACULDADE EVANGÉLICA MACKENZIE DO PARANÁ | Carolina Maria Pozzi | 4.166.924 |
| PONTIFÍCIA UNIVERSIDADE CATÓLICA DO RIO GRANDE DO SUL - PUC/RS | Leonardo Viliano Kroth | 4.216.709 |
| HOSPITAL E MATERNIDADE ANGELINA CARON | Carlos Gustavo Wing Chong Marmanillo | 4.249.225 |
| HOSPITAL SANTA IZABEL - SANTA CASA DE MISERICÓRDIA DA BAHIA / PROF DR CELSO FIGUEIRÔA | Carolina Lara Neves | 4.462.077 |
| FACULDADE DE CIÊNCIAS MÉDICAS DE MINAS GERAIS - FCM-MG | Marcus Faria Lasmar | 4.572.338 |

Footnote:

In all cases where informed consent was obtained, it was written. In some specific cases, the informed consent was waived by the local ethics committees.

Legend: IRB, Institutional Review Board; PI, principal investigator
