# Supplementary material for: High mortality among kidney transplant recipients diagnosed with coronavirus disease 2019: Results from the Brazilian multicenter cohort study
Source: PLoS One. 2021 Jul 28;16(7):e0254822. doi: 10.1371/journal.pone.0254822 (PMC8318290; doi:10.1371/journal.pone.0254822)
Supplement: S2 Table — Footnote: the attributable symptoms were defined by the local investigator. (DOCX) [file pone.0254822.s002.docx]

S2 Table. COVID-19-attributable symptoms Codebook

| **V20** | **Symptoms or signs at the first evaluation or at the COVID-19 diagnosis** | **Checkbox** |
| --- | --- | --- |
| 1 | v20_symptom___1 | Fever |
| 2 | v20_symptom___2 | Chills |
| 3 | v20_symptom___3 | Cough |
| 4 | v20_symptom___4 | Expectoration |
| 5 | v20_symptom___5 | Dyspnea |
| 6 | v20_symptom___6 | Chest pain |
| 7 | v20_symptom___7 | Coryza |
| 8 | v20_sintomas_adm___8 | Headache |
| 9 | v20_symptom___9 | Nasal congestion |
| 10 | v20_symptom___10 | Fatigue |
| 11 | v20_symptom___11 | Myalgia |
| 12 | v20_symptom___12 | Arthralgia |
| 13 | v20_symptom___13 | Nausea / vomiting |
| 14 | v20_symptom___14 | Diarrhea |
| 15 | v20_symptom___15 | Conjunctivitis |
| 16 | v20_symptom___16 | Rash |
| 18 | v20_symptom___18 | Anosmia/Hyposmia |
| 19 | v20_symptom___19 | Ageusia/Hypogeusia |
| 17 | v20_symptom___17 | Other |
| 20 | v20_symptom___20 | No Symptoms |
| **V21** | **Specify other symptom or sign:** | **Descriptive** |

**Footnote:** the attributable symptoms were defined by the local investigator.
