# Supplementary material for: High mortality among kidney transplant recipients diagnosed with coronavirus disease 2019: Results from the Brazilian multicenter cohort study
Source: PLoS One. 2021 Jul 28;16(7):e0254822. doi: 10.1371/journal.pone.0254822 (PMC8318290; doi:10.1371/journal.pone.0254822)
Supplement: S3 Table — Legend: AKI, acute kidney injury; ICU, intensive care unit; MV, mechanical ventilation; RRT, renal replacement therapy. (DOCX) [file pone.0254822.s003.docx]

S3 Table. Outcomes according to the time after transplantation

| Outcomes | Time after transplantation | | P-value |
| --- | --- | --- | --- |
|  | ≤ 1 year  N = 244 | > 1 year  N = 1,432 |  |
| Hospitalization | 174 (71.3) | 916 (64.0) | 0.031 |
| ICU requirement | 88 (36.5) | 487 (34.2) | 0.531 |
| MV requirement | 68 (28.3) | 345 (24.2) | 0.196 |
| AKI | 35 (20.8) | 207 (23.5) | 0.510 |
| RRT | 74 (30.6) | 315 (22.1) | 0.005 |
| Death | 53 (21.7) | 303 (21.2) | 0.909 |

**Legend:**

AKI, acute kidney injury; ICU, intensive care unit; MV, mechanical ventilation; RRT, renal replacement therapy.
