# Supplementary material for: High mortality among kidney transplant recipients diagnosed with coronavirus disease 2019: Results from the Brazilian multicenter cohort study
Source: PLoS One. 2021 Jul 28;16(7):e0254822. doi: 10.1371/journal.pone.0254822 (PMC8318290; doi:10.1371/journal.pone.0254822)
Supplement: S4 Table — Footnote: Missing values: BMI = 92 (5.5%); time after transplantation = 4 (0.24%); eGFR = 183 (10.9%); Recent high dose of steroids = 33 (2.0%); Recent use of Thymoglobulin = 50 (3.0%); AKI = 628 (37.4%). Legend: AKI, acute kidney injury; AZA, azathioprine; BMI, body mass index; CNI, calcineurin inhibitor; DD, deceased donor; eGFR, estimated glomerular filtration rate; IS, immunosuppressive; KAL, kidney after liver; KT, kidney transplant; LD, living donor; mTORi, mammalian target of rapamycin inhibitor; PAK, pancreas after kidney; SHK, simultaneous heart-kidney; SLK, simultaneous liver-kidney; SPK, simultaneous pancreas-kidney. (DOCX) [file pone.0254822.s004.docx]

S4 Table. Comparison between hospitalized and non-hospitalized patients

| Variable | Hospitalization | | P-value |
| --- | --- | --- | --- |
|  | No | Yes |  |
|  | N = 586 | N = 1,094 |  |
| Demographic data |  |  |  |
| Age (years) | 46.0 (38.0; 56.0) | 54.0 (44.0; 62.0) | <0.001 |
| Male sex – n (%) | 335 (57.2) | 680 (62.2) | 0.052 |
| Afro-Brazilian ethnicity – n (%) | 55 (9.4) | 136 (12.4) | 0.073 |
| BMI (kg/m^2^) | 26.5 (23.7; 29.3) | 26.4 (23.6; 29.9) | 0.303 |
| BMI > 30 kg/m^2^ – n (%) | 116 (21.3) | 262 (25.1) | 0.107 |
| Living donor | 207 (35.3) | 317 (29.0) | 0.009 |
| Time after KT (years) | 5.5 (2.1; 10.2) | 5.9 (2.3; 10.6) | 0.952 |
| Comorbidities – n (%) |  |  |  |
| *Hypertension* | 397 (67.7) | 875 (80.0) | <0.001 |
| *Diabetes* | 149 (25.4) | 422 (38.6) | <0.001 |
| *Cardiovascular* | 36 (6.1) | 170 (15.5) | <0.001 |
| *Pulmonary* | 16 (2.7) | 38 (3.5) | 0.500 |
| *Neurologic* | 5 (0.9) | 15 (1.4) | 0.486 |
| *Hepatic* | 17 (2.9) | 46 (4.2) | 0.228 |
| *Neoplasia* | 17 (2.9) | 67 (6.1) | 0.006 |
| *Autoimmune* | 21 (3.6) | 28 (2.6) | 0.300 |
| IS regimen |  |  | <0.001 |
| *CNI-AZA* | 115 (19.6) | 146 (13.3) |  |
| *CNI-MPA* | 322 (54.9) | 676 (61.8) |  |
| *CNI-mTORi* | 70 (11.9) | 87 (8.0) |  |
| *CNI-free* | 43 (7.3) | 122 (11.2) |  |
| *Other* | 36 (6.2) | 63 (5.7) |  |
| eGFR (mL/min/1.73 m^2^) | 54.9 (41.8; 71.0) | 44.3 (28.7; 61.4) | <0.001 |
| Recent high dose of steroids | 13 (2.2) | 60 (5.5) | 0.004 |
| Recent use of Thymoglobulin | 7 (1.2) | 40 (3.7) | 0.002 |
| COVID-19 characteristics |  |  |  |
| Time of symptoms | 5.0 (3.0; 9.0) | 6.0 (3.0; 9.0) | 0.061 |
| Fever | 312 (53.5) | 696 (63.7) | <0.001 |
| Chills | 154 (26.4) | 339 (31.0) | 0.056 |
| Cough | 288 (49.4) | 614 (56.2) | 0.009 |
| Dyspnea | 102 (17.5) | 510 (46.7) | <0.001 |
| Runny nose | 138 (23.7) | 153 (14.0) | <0.001 |
| Nasal congestion | 98 (16.8) | 90 (8.2) | <0.001 |
| Sore throat | 73 (12.5) | 75 (6.9) | <0.001 |
| Chest pain | 22 (3.8) | 57 (5.2) | 0.228 |
| Expectoration | 18 (3.1) | 41 (3.8) | 0.573 |
| Headache | 206 (35.3) | 198 (18.1) | <0.001 |
| Myalgia | 260 (44.6) | 415 (38.0) | 0.010 |
| Asthenia | 105 (18.0) | 210 (19.2) | 0.593 |
| Arthralgia | 29 (5.0) | 13 (1.2) | <0.001 |
| Diarrhea | 139 (23.8) | 377 (34.5) | <0.001 |
| Anosmia | 207 (35.5) | 200 (18.3) | <0.001 |
| Ageusia | 85 (14.6) | 72 (6.6) | <0.001 |
| Nausea or vomiting | 34 (5.8) | 115 (10.5) | 0.002 |
| Hypoxemia | 3 (0.5) | 206 (18.8) | <0.001 |
| AKI | 18 (7.4) | 226 (28.0) | <0.001 |

**Footnotes:**

Missing values: BMI = 92 (5.5%); time after transplantation = 4 (0.24%); eGFR = 183 (10.9%); Recent high dose of steroids = 33 (2.0%); Recent use of Thymoglobulin = 50 (3.0%); AKI = 628 (37.4%).

**Legend:**

AKI, acute kidney injury; AZA, azathioprine; BMI, body mass index; CNI, calcineurin inhibitor; DD, deceased donor; eGFR, estimated glomerular filtration rate; IS, immunosuppressive; KAL, kidney after liver; KT, kidney transplant; LD, living donor; mTORi, mammalian target of rapamycin inhibitor; PAK, pancreas after kidney; SHK, simultaneous heart-kidney; SLK, simultaneous liver-kidney; SPK, simultaneous pancreas-kidney.
