# Supplementary material for: High mortality among kidney transplant recipients diagnosed with coronavirus disease 2019: Results from the Brazilian multicenter cohort study
Source: PLoS One. 2021 Jul 28;16(7):e0254822. doi: 10.1371/journal.pone.0254822 (PMC8318290; doi:10.1371/journal.pone.0254822)
Supplement: S5 Table — Footnotes: Missing values: BMI = 92 (5.5%); time after transplantation = 4 (0.24%); eGFR = 183 (10.9%); Recent high dose of steroids = 33 (2.0%); Recent use of Thymoglobulin = 50 (3.0%); AKI = 628 (37.4%). Legend: AKI, acute kidney injury; AZA, azathioprine; BMI, body mass index; CNI, calcineurin inhibitor; DD, deceased donor; eGFR, estimated glomerular filtration rate; IS, immunosuppressive; KAL, kidney after liver; KT, kidney transplant; LD, living donor; mTORi, mammalian target of rapamycin inhibitor; PAK, pancreas after kidney; SHK, simultaneous heart-kidney; SLK, simultaneous liver-kidney; SPK, simultaneous pancreas-kidney. (DOCX) [file pone.0254822.s005.docx]

S5 Table. Comparison between survivors and non-survivors

| Variable | Survivors | Non-survivors | P-value |
| --- | --- | --- | --- |
|  | N = 1,331 | N = 349 |  |
| Demographic data |  |  |  |
| Age (years) | 49.0 (40.0; 58.0) | 58,1 (51.5; 65.3) | <0.001 |
| Male sex – n (%) | 795 (59.7) | 220 (63.0) | 0.288 |
| Afro-Brazilian ethnicity – n (%) | 156 (11.7) | 35 (10.0) | 0.429 |
| BMI (kg/m^2^) | 26.4 (23.5; 29.6) | 27.4 (24.4; 1.1) | 0.105 |
| BMI > 30 kg/m^2^ – n (%) | 288 (22.9) | 90 (27.2) | 0.120 |
| Living donor | 436 (32.8) | 88 (25.2) | 0.008 |
| Time after KT (years) | 6.9 (2.4; 11.5) | 5.5 (2.3; 10.3) | 0.015 |
| Comorbidities – n (%) |  |  |  |
| *Hypertension* | 983 (73.9) | 289 (82.8) | 0.001 |
| *Diabetes* | 410 (30.8) | 161 (46.1) | <0.001 |
| *Cardiovascular* | 125 (9.4) | 81 (23.2) | <0.001 |
| *Pulmonary* | 36 (2.7) | 18 (5.2) | 0.032 |
| *Neurologic* | 16 (1.2) | 4 (1.1) | 0.999 |
| *Hepatic* | 51 (3.8) | 12 (3.4) | 0.852 |
| *Neoplasia* | 55 (4.1) | 29 (8.3) | 0.002 |
| *Autoimmune* | 42 (3.2) | 7 (2.0) | 0.338 |
| IS regimen |  |  | 0.027 |
| *CNI-AZA* | 213 (16.0) | 48 (13.8) |  |
| *CNI-MPA* | 773 (58.1) | 225 (64.5) |  |
| *CNI-mTORi* | 139 (10.4) | 18 (5.2) |  |
| *CNI-free* | 128 (9.6) | 37 (10.6) |  |
| *Other* | 78 (5.9) | 21 (6.0) |  |
| eGFR (mL/min/1.73 m^2^) | 41.7 (27.0; 58.4) | 51.3 (35.1; 67.8) | <0.001 |
| Recent high dose of steroids | 50 (3.8) | 23 (6.6) | 0.056 |
| Recent use of Thymoglobulin | 35 (2.6) | 12 (3.4) | 0.207 |
| COVID-19 characteristics |  |  |  |
| Time of symptoms | 5.0 (3.0; 7.0) | 6.0 (3.0; 10.) | 0.030 |
| Nosocomial source of infection | 69 (5.2) | 69 (19.9) | <0.001 |
| Fever | 807 (60.8) | 201 (57.6) | 0.302 |
| Chills | 383 (28.9) | 110 (31.5) | 0.366 |
| Cough | 702 (52.9) | 200 (57.3) | 0.159 |
| Dyspnea | 390 (29.4) | 222 (63.6) | <0.001 |
| Runny nose | 253 (19.1) | 38 (10.9) | <0.001 |
| Nasal congestion | 161 (12.1) | 27 (7.7) | 0.026 |
| Sore throat | 130 (9.8) | 18 (5.2) | 0.009 |
| Chest pain | 65 (4.9) | 14 (4.0) | 0.580 |
| Expectoration | 41 (3.1) | 18 (5.2) | 0.089 |
| Headache | 363 (27.4) | 41 (11.7) | <0.001 |
| Myalgia | 575 (43.3) | 100 (28.7) | <0.001 |
| Asthenia | 268 (20.2) | 47 (13.5) | 0.005 |
| Arthralgia | 41 (3.1) | 1 (0.3) | 0.005 |
| Diarrhea | 410 (30.9) | 106 (30.4) | 0.902 |
| Anosmia | 362 (27.3) | 45 (12.9) | <0.001 |
| Ageusia | 141 (10.6) | 16 (4.6) | 0.001 |
| Nausea or vomiting | 127 (9.6) | 22 (6.3) | 0.071 |
| Hypoxemia | 104 (7.8) | 105 (30.1) | <0.001 |
| AKI | 158 (19.5) | 86 (35.8) | <0.001 |

**Footnotes:**

Missing values: BMI = 92 (5.5%); time after transplantation = 4 (0.24%); eGFR = 183 (10.9%); Recent high dose of steroids = 33 (2.0%); Recent use of Thymoglobulin = 50 (3.0%); AKI = 628 (37.4%).

**Legend:**

AKI, acute kidney injury; AZA, azathioprine; BMI, body mass index; CNI, calcineurin inhibitor; DD, deceased donor; eGFR, estimated glomerular filtration rate; IS, immunosuppressive; KAL, kidney after liver; KT, kidney transplant; LD, living donor; mTORi, mammalian target of rapamycin inhibitor; PAK, pancreas after kidney; SHK, simultaneous heart-kidney; SLK, simultaneous liver-kidney; SPK, simultaneous pancreas-kidney.
