# Supplementary material for: High mortality among kidney transplant recipients diagnosed with coronavirus disease 2019: Results from the Brazilian multicenter cohort study
Source: PLoS One. 2021 Jul 28;16(7):e0254822. doi: 10.1371/journal.pone.0254822 (PMC8318290; doi:10.1371/journal.pone.0254822)
Supplement: Attachment — Submitted filename: Response_to_Reviewers.docx [file pone.0254822.s007.docx]

Dear Academic Editor

Dr. Giuseppe Remuzzi

On behalf of authors, we thank the opportunity to resubmit to **PLOS ONE** the revised version of our manuscript entitled **“High mortality among kidney transplant recipients diagnosed with coronavirus disease 2019: Results from the Brazilian multicenter cohort study.”** (PONE-D- 21-05548).

Your comments as well as the comments of the reviewers were crucial to improve our manuscript and all issues raised by you are following addressed.

Lúcio R. Requião-Moura

**Review**

**Academic editor**

The manuscript focuses on a topic of current interest. The study, however, presents several shortcomings that the authors need to address. To mention some of them:

| **From the editor:** | **Author comments:** |
| --- | --- |
| i) unclear when the informed consent was obtained from all patients | Thank you for the opportunity to clarify this statement.  The informed consent was obtained to have access to the patients’ medical records. As the study was a retrospective analysis, in cases of exemption, it followed specific national legislations, the local Institutional Review Board (IRB) recommendations, and the guidelines of the Declaration of Helsinki.  It was clarified in the methods section, as following:  “*Informed consent or its exemption followed specific national legislations, the local Institutional Review Board (IRB) recommendations, and the guidelines of the Declaration of Helsinki.”* |
| ii) unclear the criteria for diagnosis of COVID-19 infection | The COVID-19 diagnosis was considered if patients presented at least one COVID-19-attributable symptom associated with a positive test. A codebook with the COVID-19-attributable symptoms was included in the supplementary material.  The tests to confirm the COVID-19 diagnosed were (one of them, at least): polymerase chain reaction, serology, or viral antigen detection. The diagnosis based on serology was retrospectively performed in patients who had had attributable symptoms (4 weeks after the beginning of the symptoms, in average).  It was clarified in the methods section, as following:  “*The diagnosis was considered only in patients who presented at least one COVID-19-attributable symptom associated with a positive result in any one of the following tests: polymerase chain reaction (PCR), serology, or viral antigen detection. A codebook with the description of the attributable symptoms is presented in the supplementary table 1. The diagnosis based on serology was retrospectively performed in patients who had had attributable symptoms. The attributable symptoms were defined by the local investigator*”. |
| iii) need to elaborate the means by which the symptoms were recorded on the REDcap system by the transplant/primary care physicians | A codebook with the COVID-19-attributable symptoms was included in the supplementary material (supplementary table 1). The investigators could check one or more symptoms in the list using a check box. Symptoms or signs not listed in the codebook could be reported in a descriptive field. |
| iv) need to clarify when the serology test was done in the 15.3% of patients who were diagnosed by means of serology rather than in a PCR | The diagnosis based on serology was retrospectively performed in patients who had had attributable symptoms. The time between the beginning of symptoms and the serology was 26.0 (9.0; 55.0) days.  In the current version of the manuscript, we clarified this information in the methods sections and the time between the beginning of symptoms and serology was included in the results.  In the methods:  “*The diagnosis based on serology was retrospectively performed in patients who had had attributable symptoms”.*  In the results:  “*Among patients who had the diagnosis based on serology, the time between the COVID-19 attributable symptoms beginning and serology was 26.0 (9.0; 55.0) days*”. |
| v) need to perform multivariate analysis of the outcomes including age and frailty | Thank you for this suggestion.  We performed multivariate analysis for the probability of hospitalization and death (comments in the topic xii). Unfortunately, information about frailty was not available.  The results of the multivariable analysis were depicted in two tables in the current version of the manuscript: 4 and 5. |
| vi) unclear what percent of the transplant population in Brazil is Afro-Brazilian | There are no official data about race among transplanted patients in Brazil. Moreover, Brazil is a multiracial country, and the definition of race is self-reported, what has more correlation with the social identity than a biological background.  In a recent multicenter study (n=3,992) carried out to investigate the incidence of delayed graft function after kidney transplant performed with deceased donors the frequency of Afro-Brazilian was 13.7% (DOI: doi:10.1111/tri.13865).  Additionally, a previous study that evaluated 10,000 kidney transplants performed in a Brazilian single center, the frequency of Afro-Brazilian was 23% (DOI: 10.1007/s40620-017-0379-9).  These frequencies are similar those that was observed in the present study. |
| vii) unclear whether they have data on transplant induction therapy (lymphocyte depleting versus non-depleting or no induction) | Data on transplant induction therapy were not available.  Actually, we presented the immunosuppressive regimen that patients were using at the COVID-19 diagnosis. Of note, the time interval between transplantation and COVID-19 infection was 5.9 years, however we consider that the lack of this information could be high light as a limitation. We included a comment about this limitation in the discussion of the current version of the manuscript.  In respect to immunosuppression, we were especially interested in the use of high doses of steroids or lymphocyte depleting antibody to treat acute rejection until 3 months from the COVID-19 diagnosis, considered as recent use.  Recent use of high dose of steroids was recorded for 4.3% (n=73) patients, whereas lymphocyte depleting antibody was recorded for 2.8% (n=47).  These results were embodied in the results, as following:  “*Use of high dose of steroids and lymphocyte depleting antibody to treat acute rejection until 3 months before the COVID-19 diagnosis were recorded for 4.3% (n=73) and 2.8% (n=47), respectively*.” |
| viii) need to provide the time between symptoms and hospitalization in those hospitalized | The time between symptoms and hospitalization was 7.0 (4.0; 10.0) days. This information was included in the results of the current version of the manuscript, as following:  “*About two-thirds of the patients (65.1%) were hospitalized for clinical management, 7.0 (4.0; 10.0) days after the beginning of symptoms*.” |
| ix) unclear whether nosocomial infections occur at the time of transplantation or while admitted for another cause | Thank you for the opportunity to clarify this information: 137 patients had the nosocomial infection as the source of infection. Among them, in 16 (11.7%) the infection occurred during the hospitalization when the transplantation was performed. We embodied this information in the current version of the manuscript. |
| x) useful to have information on socioeconomic status in the current study | The socioeconomic status was not available in the present study. We agree that it is a very important point, and it should be addressed in future investigations. We are including this statement in the discussion. |
| xi) need to provide the outcome in patients transplanted early (<1 year) vs late (>1 year) | Thank you for this suggestion.  The outcomes were compared according to the time of transplantation, which was dichotomized in $\leq$1 year and >1 years. These results were included in the supplementary table 2 in the current version of the manuscript.  The hospitalization rate was higher among patients who had the time after transplantation shorter than 1 year (71.3% vs. 64.0%, P=0.031), as well as the requirement for RRT (30.6% vs. 22.1, P=0.005).  The following sentence was included in the results in the current version:  “*The outcomes were compared according to the time of transplantation: longer or shorter than 1 year (supplementary table 2). The hospitalization rate was higher among patients who had the time after transplantation shorter than 1 year (71.3% vs. 64.0%, P=0.031), as well as the requirement for RRT (30.6% vs. 22.1, P=0.005).”* |
| xii) need to perform a comparison between hospitalized and non-hospitalized, as well as survivors vs non-survivors | Thank you for this suggestion.  We compared the variables according to the hospitalization (yes vs. no) and death (survivors vs. non-survivors). The continuous variables were compared by Mann-Whitney U test and the categorical were compared by *X^2^* or Fisher test. We are including these results in the current version of the manuscript.  In the supplementary table 3 is shown the comparison between patients who were hospitalized versus those who were not. The variables that reached a P-value $\leq$ 0.10 in the univariable comparison were selected for the multivariable modeling. The multivariable analysis was performed by the binary logistic regression. The final results are shown in the table 4.  In summary, the variables related with the probability of hospitalization were age, hypertension, previous cardiovascular disease, recent use of high dose of steroid, and fever, dyspnea, diarrhea, and nausea/vomiting as COVID-19 symptoms. On the other hand, the variables that reduced the probability of hospitalization were time of COVID-19 symptoms, and nasal congestion, headache, arthralgia and anosmia as COVID-19 symptoms.  In the supplementary table 4 is shown the comparison between patients who survived versus those who did not. Similarly, the variables that reached a P-value $\leq$ 0.10 in the univariable comparison were selected for the multivariable modeling. The multivariable analysis was performed by the binary logistic regression. The final results are shown in the table 5.  The variables related with the probability of death within 90 days after COVID-19 were age, time after transplantation, presence of hypertension, previous cardiovascular disease, use of tacrolimus and mycophenolate as the maintenance immunosuppression, recent use of high dose of steroids, and dyspnea as COVID-19 symptom. On the other hand, the variables that reduced the risk of death were time of symptoms, and headache and anosmia as COVID-19 symptoms. |
| xiii) unclear whether any patients have a kidney biopsy and if there were any episodes of rejection | Unfortunately, information about kidney biopsy after the COVID-19 diagnosis was not available. We consider that this lack of information is a limitation to understand the results about acute kidney injury, and we included this limitation in the discussion. |

Reviewer #1: The study on mortality in kidney transplant recipients with coronavirus disease from the Brazilian transplant centres is a large cohort analysed for the effects of COVID-19 infection in kidney transplant recipients. The study shows our mortality of 21% would you creases with hospital admission and ICU admission to 31 and 76% respectively.

The study is well conducted with a large patient population from 35 Centres in Bazil.

| **Reviewer #1 comments:** | **Author comments:** |
| --- | --- |
| The informed consent was obtained from all patients but perhaps it’s not clear when the content was obtained as the study was a retrospective analysis | Thank you for the opportunity to clarify this statement.  The informed consent was obtained to have access to the patients’ medical records. As the study was a retrospective analysis, in cases of exemption, it followed specific national legislations, the local Institutional Review Board (IRB) recommendations, and the guidelines of the Declaration of Helsinki.  It was clarified in the methods section, as following:  “*Informed consent or its exemption followed specific national legislations, the local Institutional Review Board (IRB) recommendations, and the guidelines of the Declaration of Helsinki.”* |
| The criteria for diagnosis of COVID-19 infection is not very clear. The study mentioned diagnosis was according to the clinical criteria for each centre. However the definition could’ve been different in the different centres which requires a mention in the article. | Thank you for this comment.  The criteria for diagnosis was standardized for all centers, and it was based on two aspects: the test and COVID-19-attributable symptoms. For all patients, the COVID-19 diagnosis was considered if patients presented at least one COVID-19-attributable symptom associated with a positive test. A codebook with the COVID-19-attributable symptoms was included in the supplementary material to clarify how the information was collected.  The tests to confirm the COVID-19 diagnosed were (one of them, at least): polymerase chain reaction, serology, or viral antigen detection. The diagnosis based on serology was retrospectively performed in patients who had had attributable symptoms (4 weeks after the beginning of the symptoms, in average).  It was clarified in the methods section, as following:  “*The diagnosis was considered only in patients who presented at least one COVID-19-attributable symptom associated with a positive result in any one of the following tests: polymerase chain reaction (PCR), serology, or viral antigen detection. A codebook with the description of the attributable symptoms is presented in the supplementary table 1. The diagnosis based on serology was retrospectively performed in patients who had had attributable symptoms. The attributable symptoms were defined by the local investigator”* |
| The authors should be congratulated for collecting data on symptoms. However the authors could elaborate the means by which the symptoms were recorded on the REDcap system by the transplant/primary care physicians. | A codebook with the COVID-19-attributable symptoms was included in the supplementary material (supplementary table 1). The investigators could check one or more symptoms in the list using a check box. Symptoms or signs not listed in the codebook could be reported in a descriptive field. |
| The author should also clarify when was the serology test done in the 15.3% of the patients who were diagnosed by means of serology rather than are in a PCR. | It is a very important point. Thank you for your comment.  The diagnosis based on serology was retrospectively performed in patients who had had attributable symptoms. The time between the beginning of symptoms and the serology was 26.0 (9.0; 55.0) days.  In the current version of the manuscript, we clarified this information in the methods sections and the time between the beginning of symptoms and serology was included in the results.  In the methods:  “*The diagnosis based on serology was retrospectively performed in patients who had had attributable symptoms”.*  In the results:  “*Among patients who had the diagnosis based on serology, the time between the COVID-19 attributable symptoms beginning and serology was 26.0 (9.0; 55.0) days*”. |
| The authors have emphasised the role of acute kidney injury and RRT on outcome. A multivariate analysis of the outcomes including age and frailty possible would be very useful. | Thank you for your suggestion.  We performed multivariate analysis for the probability of hospitalization and death, as it was suggested by the Academic editor. Unfortunately, information about frailty was not available.  The results of the multivariable analysis were depicted in two tables in the current version of the manuscript: 4 and 5.  In summary, the variables related with the probability of hospitalization were age, hypertension, previous cardiovascular disease, recent use of high dose of steroid, and fever, dyspnea, diarrhea, and nausea/vomiting as COVID-19 symptoms. On the other hand, the variables that reduced the probability of hospitalization were time of COVID-19 symptoms, and nasal congestion, headache, arthralgia and anosmia as COVID-19 symptoms.  Additionally, the variables related with the probability of death within 90 days after COVID-19 were age, time after transplantation, presence of hypertension, previous cardiovascular disease, use of tacrolimus and mycophenolate as the maintenance immunosuppression, recent use of high dose of steroids, and dyspnea as COVID-19 symptom. On the other hand, the variables that reduced the risk of death were time of symptoms, and headache and anosmia as COVID-19 symptoms. |

Reviewer #2: The authors of the manuscript “High mortality among kidney transplant recipients diagnosed with coronavirus disease 2019: Results from the Brazilian multicenter cohort study” should be commended for performing the largest cohort study of COVID19 in kidney transplant recipients published to date. Besides being the largest, the authors were able to capture migratory patterns of infection within Brazil and nicely explore the continuum of infection from diagnosis to 90 days. In addition, informed consent was obtained. This allows insight into both outpatient and inpatient mortality rates among other insights, a feature lacking in several other published manuscripts on the subject. Although I am happy with the manuscript as is, I wonder if the authors could explore a few other questions of interest, as their large database may allow further analysis.

| **Reviewer #2 comments:** | **Author comments:** |
| --- | --- |
| I notice only 11.4% of patients were Afro-Brazilian. What percent of the transplant population in Brazil is Afro-Brazilian? | In a recent multicenter study (n=3,992) carried out to investigate the incidence of delayed graft function after kidney transplant performed with deceased donors the frequency of Afro-Brazilian was 13.7% (DOI: doi:10.1111/tri.13865).  Additionally, a previous study that evaluated 10,000 kidney transplants performed in a Brazilian single center, the frequency of Afro-Brazilian was 23% (DOI: 10.1007/s40620-017-0379-9).  These frequencies are similar those that was observed in the present study. |
| Do the authors have data on transplant induction therapy? Lymphocyte depleting versus non-depleting or no induction. | Data on transplant induction therapy were not available.  Actually, we presented the immunosuppressive regimen that patients were using at the COVID-19 diagnosis. Of note, the time interval between transplantation and COVID-19 infection was 5.9 years, however we consider that the lack of this information could be high light as a limitation. We included a comment about this limitation in the discussion of the current version of the manuscript.  In respect to immunosuppression, we were especially interested in the use of high doses of steroids or lymphocyte depleting antibody to treat acute rejection until 3 months from the COVID-19 diagnosis, considered as recent use.  Recent use of high dose of steroids was recorded for 4.3% (n=73) patients, whereas lymphocyte depleting antibody was recorded for 2.8% (n=47).  These results were embodied in the results, as following:  “*Use of high dose of steroids and lymphocyte depleting antibody to treat acute rejection until 3 months before the COVID-19 diagnosis were recorded for 4.3% (n=73) and 2.8% (n=47), respectively*.” |
| What was the time between symptoms and hospitalization in those hospitalized? This data would be useful for clinicians to know when patients are at the highest risk for hospitalization. | Thank you for yuor very important suggestion.  The time between symptoms and hospitalization was 7.0 (4.0; 10.0) days. This information was included in the results of the current version of the manuscript, as following:  “*About two-thirds of the patients (65.1%) were hospitalized for clinical management, 7.0 (4.0; 10.0) days after the beginning of symptoms*.” |
| Nosocomial infections were high. Did these occur at the time of transplantation or while admitted for another cause? | Thank you for the opportunity to clarify this information: 137 patients had the nosocomial infection as the source of infection. Among them, in 16 (11.7%) the infection occurred during the hospitalization when the transplantation was performed. We embodied this information in the current version of the manuscript. |
| The authors mention deep social inequality in Brazil. Do they have any information on socioeconomic status in the current study? | The socioeconomic status was not available in the present study. We agree that it is a very important point, and it should be addressed in future investigations. We are including this statement in the discussion. |
| As the authors have a good number of patients perhaps they analyze the following: a. Outcomes in patients transplanted early (<1 year) vs late (>1 year) b. Outcomes in patients without patient reduction in immunosuppression versus no change. It would be interesting to know if early reduction in immunosuppression can help avoid hospital admission or perhaps it makes no difference. c. Can the authors perform a comparison between hospitalized and non-hospitalized, as well as survivors vs. non-survivors. Were there any other characteristics besides AKI that predicted a higher risk for adverse outcome (for example age, race, co-morbid conditions, regions, or times of disease)? d. Did any patients have a kidney biopsy? Were there any episodes of rejection? | Point a:  Thank you for your suggestion.  The outcomes were compared according to the time of transplantation, which was dichotomized in $\leq$1 year and >1 years. These results were included in the supplementary table 2 in the current version of the manuscript.  The hospitalization rate was higher among patients who had the time after transplantation shorter than 1 year (71.3% vs. 64.0%, P=0.031), as well as the requirement for RRT (30.6% vs. 22.1, P=0.005).  The following sentence was included in the results in the current version:  “*The outcomes were compared according to the time of transplantation: longer or shorter than 1 year (supplementary table 2). The hospitalization rate was higher among patients who had the time after transplantation shorter than 1 year (71.3% vs. 64.0%, P=0.031), as well as the requirement for RRT (30.6% vs. 22.1, P=0.005).”*  Point b:  We totally agree this is a very relevant point. It would be interesting to seek if early management could impact in the outcomes, such as hospitalization. However, the time when the immunosuppression was reduced or withdrawn was not available.  Point c:  Thank you for this suggestion. We compared the variables according to the hospitalization (yes vs. no) and death (survivors vs. non-survivors). The continuous variables were compared by Mann-Whitney U test and the categorical were compared by *X^2^* or Fisher test. We are including these results in the current version of the manuscript.  In the supplementary table 3 is shown the comparison between patients who were hospitalized versus those who were not. The variables that reached a P-value $\leq$ 0.10 in the univariable comparison were selected for the multivariable modeling. The multivariable analysis was performed by the binary logistic regression. The final results are shown in the table 4.  In summary, the variables related with the probability of hospitalization were age, hypertension, previous cardiovascular disease, recent use of high dose of steroid, and fever, dyspnea, diarrhea, and nausea/vomiting as COVID-19 symptoms. On the other hand, the variables that reduced the probability of hospitalization were time of COVID-19 symptoms, and nasal congestion, headache, arthralgia and anosmia as COVID-19 symptoms.  In the supplementary table 4 is shown the comparison between patients who survived versus those who did not. Similarly, the variables that reached a P-value $\leq$ 0.10 in the univariable comparison were selected for the multivariable modeling. The multivariable analysis was performed by the binary logistic regression. The final results are shown in the table 5.  The variables related with the probability of death within 90 days after COVID-19 were age, time after transplantation, presence of hypertension, previous cardiovascular disease, use of tacrolimus and mycophenolate as the maintenance immunosuppression, recent use of high dose of steroids, and dyspnea as COVID-19 symptom. On the other hand, the variables that reduced the risk of death were time of symptoms, and headache and anosmia as COVID-19 symptoms.  Point d:  Unfortunately, information about kidney biopsy after the COVID-19 diagnosis was not available. We consider that this lack of information is a limitation to understand the results about acute kidney injury, and we included this limitation in the discussion. |
